# A bio‐ecological model for early screening of developmental coordination disorder

**DOI:** 10.1111/dmcn.70000

**Published:** 2025-10-03

**Authors:** Xiaotian Dai, Tai Ren, Gareth Williams, Gary Jones, Fei Li, Wenchong Du, Jing Hua

**Affiliations:** ^1^ Shanghai Key Laboratory of Maternal Fetal Medicine, Shanghai First Maternity and Infant Hospital, School of Medicine, Tongji University Shanghai China; ^2^ NTU Psychology, School of Social Sciences, Nottingham Trent University Nottingham UK; ^3^ Shanghai Key Laboratory of Children's Environmental Health, Xinhua Hospital, Shanghai Jiao Tong University School of Medicine Shanghai China; ^4^ School of Social Sciences, Nottingham Trent University Nottingham UK

## Abstract

**Aim:**

To develop and externally validate a bio‐ecological model for early screening of developmental coordination disorder (DCD) using maternal and environmental risk factors from electronic health records, aimed at improving early detection in children under 5 years.

**Method:**

This was a prospective study that examined data from 150 948 preschool children in China. Perinatal and sociodemographic predictors were integrated using logistic regression and random forest algorithms. The model was internally validated on split training and testing subsets and externally validated on an independent clinical sample of 1359 children aged 3 to 10 years, including confirmed diagnoses of DCD. Model performance was evaluated using the area under the curve (AUC), sensitivity, specificity, and accuracy.

**Results:**

In the group aged 3 to 5 years, the model achieved an AUC of 0.70, sensitivity of 71.43%, accuracy of 77.61%, and specificity of 78.00%. In the group aged 6 to 10 years, performance was moderate (AUC = 0.58; sensitivity = 54.88%; accuracy = 61.50%; specificity = 62.28%).

**Interpretation:**

This bio‐ecological model offers a scalable, cost‐effective tool to support the early identification of DCD using electronic health record data. It performs well in early childhood and maintains moderate accuracy in older children, supporting its utility for longer‐term risk prediction. The model could enhance existing screening systems by enabling earlier triage and intervention. Further validation across diverse health care settings is warranted.

## Graphical abstract

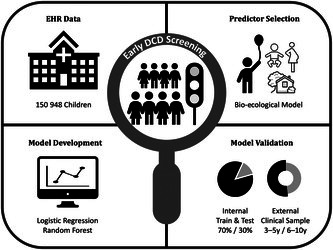

**This original article is commented by Khan et al. on page** 740 **of this issue.**

Plain language summary: https://onlinelibrary.wiley.com/doi/10.1111/dmcn.70069

**Abbreviations**
AUC: area under the curve
DCD: developmental coordination disorder
EHR: electronic health record

**What this paper adds**

- The bio‐ecological framework offers a comprehensive approach to developmental coordination disorder screening.
- Internal and external validation across different age groups confirms the model's robustness and applicability.
- Screening accuracy is enhanced in early childhood (3–5 years) and moderate accuracy is maintained in older children (6–10 years).
- Predictors that are routinely available in clinical and community health care records makes the model practical and accessible for integration into existing health care systems.

Developmental coordination disorder (DCD) is a common and chronic neurodevelopmental condition marked by significant motor coordination difficulties that cannot be explained by intellectual disability, neurological conditions, or other medical disorders. These motor impairments interfere with daily functioning and academic performance, and are frequently associated with adverse outcomes in mental health, self‐esteem, social participation, and family dynamics.^1^ DCD commonly persists into adolescence and adulthood, resulting in long‐term functional limitations and contributing to substantial societal and economic burdens, particularly within health care and educational systems.^2^, ^3^ Globally, DCD presents a significant public health challenge, affecting approximately 5% to 6% of school‐age children.^1^ Prevalence varies across continents, with 4% in Asia, 2% in Europe, and 6% in North America, depending on the diagnostic criteria, assessment tools, and cultural context.^1^ In China, emerging evidence suggests a potentially higher prevalence, with some regional studies reporting rates between 7% and 10%.^4^ Despite the prevalence of DCD, the early detection of DCD poses considerable difficulties in child health care systems: children with DCD often go unrecognized in the early years because of the subtle onset of symptoms and marked variability in early motor development among young children.^1^, ^5^ These factors significantly impede the timely diagnosis and intervention that are critical for improving long‐term outcomes.

Historically, the aetiology of DCD has been elusive, with relatively few risk factors consistently identified. However, recent advancements in research have begun to more effectively clarify the array of risk factors associated with DCD.^6^ Among these, male sex and preterm birth have emerged as the most significant predictors,^7^ with males being affected two to seven times more often than females, and children born preterm showing double the risk compared to those born at term.^1^, ^7^ Additionally, a range of factors spanning the prenatal, perinatal, and neonatal stages, and broader environmental influences, are also critical.^6^ For instance, independent risk factors, such as placenta previa and placental abruption, have been linked to DCD;^6^ low socioeconomic status within families has been identified as a contributing factor to the likelihood of developing DCD.^8^ These findings support the multifactorial nature of DCD, advocating for a bio‐ecological model that highlights the dynamic interplay of biological characteristics and nested environmental contexts, ranging from immediate family to broader societal systems.^9^ These dynamic interplays have fostered a hybrid, multiconstraint understanding of DCD, informing current clinical practice.^1^, ^10^

Early identification of children at risk for DCD is crucial to ensure timely intervention and to minimize the long‐term functional and psychosocial impacts. The current process in most health care systems typically begins with an initial screening phase to identify children at higher risk of DCD, followed by comprehensive diagnostic evaluation by trained professionals. While parent‐reported observations remain a vital and irreplaceable source of information in developmental screening, their reliability in large‐scale screening contexts can be influenced by factors such as access to standardized tools, differences in health literacy, and variation in expectations around motor development.^1^ Also, co‐occurring disorders such as autism spectrum disorder and attention‐deficit/hyperactivity disorder are common among children with motor coordination difficulties and can complicate early recognition. These limitations may result in delays in recognizing motor coordination difficulties, contributing to underdiagnosis and unmet needs among affected children.^11^, ^12^ Consequently, DCD remains a widely unrecognized condition, that is underdiagnosed and underrepresented in early care systems.^13^ To support existing identification systems, there is a need for scalable, data‐driven approaches that can complement behavioural assessments and facilitate earlier identification, particularly in community or primary care settings.

Despite growing interest in early risk prediction for neurodevelopmental conditions, few studies have developed population‐based risk algorithms specifically for DCD; even fewer have externally validated these models using routinely collected health data. This gap limits the scalability and clinical utility of early screening efforts. In response, our study aimed to develop a population‐based, data‐driven risk algorithm for DCD that serves as a pre‐screening tool to enhance the effectiveness and efficiency of the subsequent screening and diagnostic processes in the existing health care framework. Grounded in the bio‐ecological model^9^ and the International Classification of Functioning, Disability, and Health framework^14^ of DCD, this algorithm uses routinely collected health and demographic data from electronic health records to identify children at higher risk of DCD. Unlike traditional identification approaches that focus on motor performance once concerns arise, our model enables early risk stratification based on predisposing factors, such as perinatal complications, family structure, and maternal health, before motor difficulties are formally recognized or reliably observed, allowing for risk stratification in non‐clinical settings, such as communities, schools, and families. Initially developed and tested using data from children aged 3 to 5 years, the algorithm's effectiveness and generalizability were further validated through internal and external samples, including clinically diagnosed cases across broader age ranges (3–10 years). This dual‐stage validation ensures the algorithm's capability to detect DCD across several developmental stages and adapt to potential shifts in risk factor relevance and motor performance visibility as children grow. Importantly, this tool is not intended to replace clinical assessments or caregiver insights, but to support existing systems by enhancing the efficiency and equity of early identification. By prioritizing follow‐up for those most at risk, the model offers a scalable, cost‐effective complement to current screening practices and holds promise for streamlining diagnostic pathways and improving developmental outcomes.

## METHOD

Our methods are based on established medical probability and recommendations of the ‘Transparent Reporting of a multivariable prediction model for Individual Prognosis Or Diagnosis‐Artificial Intelligence’ statement,^15^ and the guidance provided by the model card framework.^16^ We developed the predictive model with a large population‐based sample and validated it independently in two external clinical samples of different age groups.

A summary model card, detailing the intended use, input features, validation results, and limitations, is provided in Appendix S1, in line with current recommendations for clinical prediction tools.

### Study setting and design

Data from the Chinese National Cohort of Motor Development was used to develop a risk prediction model for DCD outcomes in childhood. The Chinese National Cohort of Motor Development was designed to explore neurodevelopment and other health outcomes in Chinese preschool children. Data were collected from April 2018 to December 2019 across 2403 mainstream kindergartens from 551 cities in China. Participants were recruited using a cluster sampling plan that encompassed all administrative districts in mainland China. Data were managed centrally and supervised using an electronic health record system. Additional details about the cohort design, sampling procedures, and data quality management are available in previously published studies.^17^, ^18^ Children aged 3 to 5 years in the Chinese National Cohort of Motor Development study were randomly divided into two subsets: 70% in a training set and the remaining 30% in a testing set.

For external validation, the model's diagnostic and prognostic performance was tested using data from children aged 3 to 5 years, extrapolating to a sample of children aged between 6 years and 10 years. The validation process used a nationally representative sample,^19^ achieved through a stratified sampling approach, which was designed based on the National Census data for stratification according to geographical region, age, sex, and socioeconomic status. A comprehensive clinical evaluation was conducted for each participant, including detailed clinical assessments, to confirm a diagnosis of DCD according to established clinical criteria.

Ethical approval was obtained from the Ethics Committee of the Shanghai First Maternity and Infant Hospital (no. KS18156) and the Institutional Review Board of the National Key Laboratory of Cognitive Neuroscience and Learning, Beijing Normal University. Written informed consent was obtained from the parents. All data collected were kept confidential and only accessible to the registered researchers.

### Study participants

The model development phase used a population‐based sample of 150 948 children aged 3 to 5 years.^8^, ^18^ The external validation phase included a total of 1359 children, with 585 aged 3 to 5 years and 774 aged 6 to 10 years. These figures reflect all eligible participants who met the study's predefined criteria: normal intelligence; no severe visual, hearing, or other developmental impairments as assessed before kindergarten (e.g. cerebral palsy); and caregivers able to complete the online questionnaire. No further exclusions were applied.

### Assessment and definition of developmental coordination disorder outcomes

In the generating sample, children's motor coordination impairment was assessed using the Little Developmental Coordination Disorder Questionnaire,^20^ a parent‐filled questionnaire specifically adapted for children aged 3 to 5 years and approved to have satisfying validity and reliability in the Chinese population.^21^ Children scoring below the threshold of 15% on the Little Developmental Coordination Disorder Questionnaire and who had no reported medical condition that would exclude them from a diagnosis of DCD, were classified as having possible DCD.

In the external validation sample, licensed paediatricians or psychologists conducted comprehensive clinical assessments on all the children to make a diagnosis of DCD according to the criteria of the Diagnostic and Statistical Manual of Mental Disorders, Fifth Edition. All children diagnosed with DCD met these criteria and exhibited scores below the 15th centile on the Movement Assessment Battery for Children, Second Edition, which is an established tool for assessing the motor impairments associated with DCD.^1^ Children with potential comorbid conditions, such as intellectual disability, autism spectrum disorder, or attention‐deficit/hyperactivity disorder, were excluded based on clinical judgement and developmental history.

### Predictor selection

**FIGURE 1:**
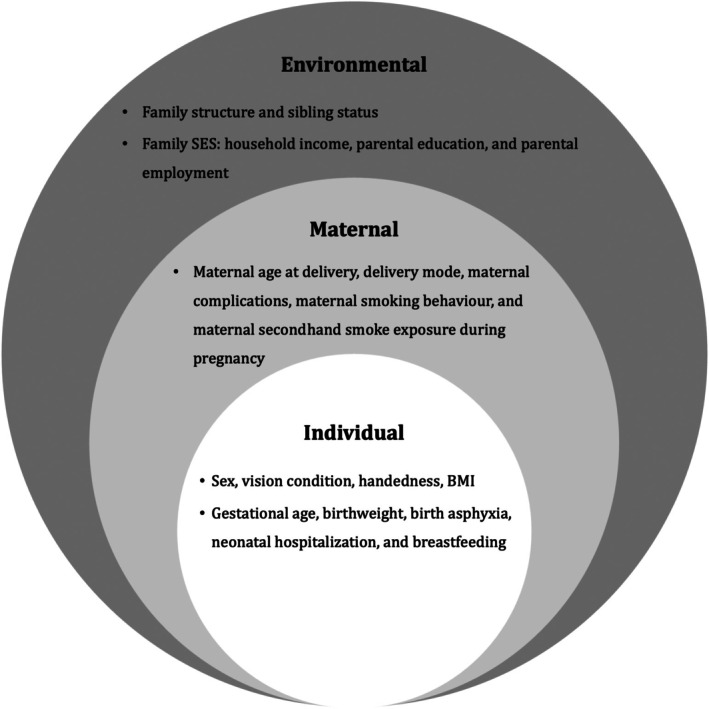
Selection of candidate predictors based on ecological theory and a literature review. Abbreviations: BMI, body mass index; SES, socioeconomic status.

The selection of candidate predictors was guided by both theoretical and clinical insights into DCD. All predictors were identified a priori, guided by the bio‐ecological model^9^ and the International Classification of Functioning, Disability, and Health framework of DCD.^14^ These models emphasize the interplay of biological, individual, and environmental factors in shaping motor development and were used to ensure comprehensive coverage of the relevant risk domains. To strengthen clinical relevance, we also drew on findings from a recent scoping review that synthesized 25 years of research on early‐life risk factors for DCD, encompassing sociodemographic, prenatal, perinatal, and postnatal domains.^7^ Only variables with strong theoretical justification or prior empirical support were retained. These candidate predictors were systematically mapped onto the conceptual frameworks to ensure alignment between model structure and established risk pathways. Twenty‐one variables were selected for inclusion in model development. To reduce the risk of misclassification, we also included task‐related variables, such as vision status, handedness, and body mass index (BMI), which may confound motor assessments by mimicking or masking coordination difficulties (Figure 1; see Table S1 for the definitions of variables and reference levels).

### Statistical analysis

All the analyses for model development and validation were conducted in R v4.3.3 (R Foundation for Statistical Computing, Vienna, Austria).

### Model development and feature contribution to prediction

We developed the model using a regression approach with backward stepwise selection because of its clinical interpretability and transportability. This was performed using the step function in R, which iteratively removes the least significant predictors based on the Akaike information criterion.

Feature contributions to prediction were quantified with a random forest algorithm. Factor significance was measured using mean decrease impurity (Gini importance), with higher values indicating greater importance in predicting DCD.

### Internal and external validation

For internal validation, a receiver operating characteristic curve analysis was used on the training set to identify the area under the curve (AUC) and the optimal cut‐off for detecting children at high risk of DCD. The optimal cut‐off was determined using Youden's J statistic to maximize the model's discriminative ability. The AUC was also assessed in the testing set for further internal validation.

For external validation, cases diagnosed with DCD were used to assess the model's performance across different age subsets. Predictions above the cut‐off were classified as positive (DCD); those below it were classified as negative (typical development). Classification outcomes were used to calculate the AUC and were categorized into true and false positives, and true and false negatives. Metrics such as sensitivity, accuracy, and specificity were reported to evaluate the model's external validation performance.

## RESULTS

For model development, 150 948 children aged 3 to 5 years were randomly divided into a training set (*n* = 105 664; 47% female) and an internal testing set (*n* = 45 284; 47% female). Two external validation sets were used: external set 1 included 585 children aged 3 to 5 years (51% female) and external set 2 included 774 children aged 6 to 10 years (47% female). The general characteristics of participants in each data set can be found in Table S2.

### Development of the risk prediction algorithm

**FIGURE 2:**
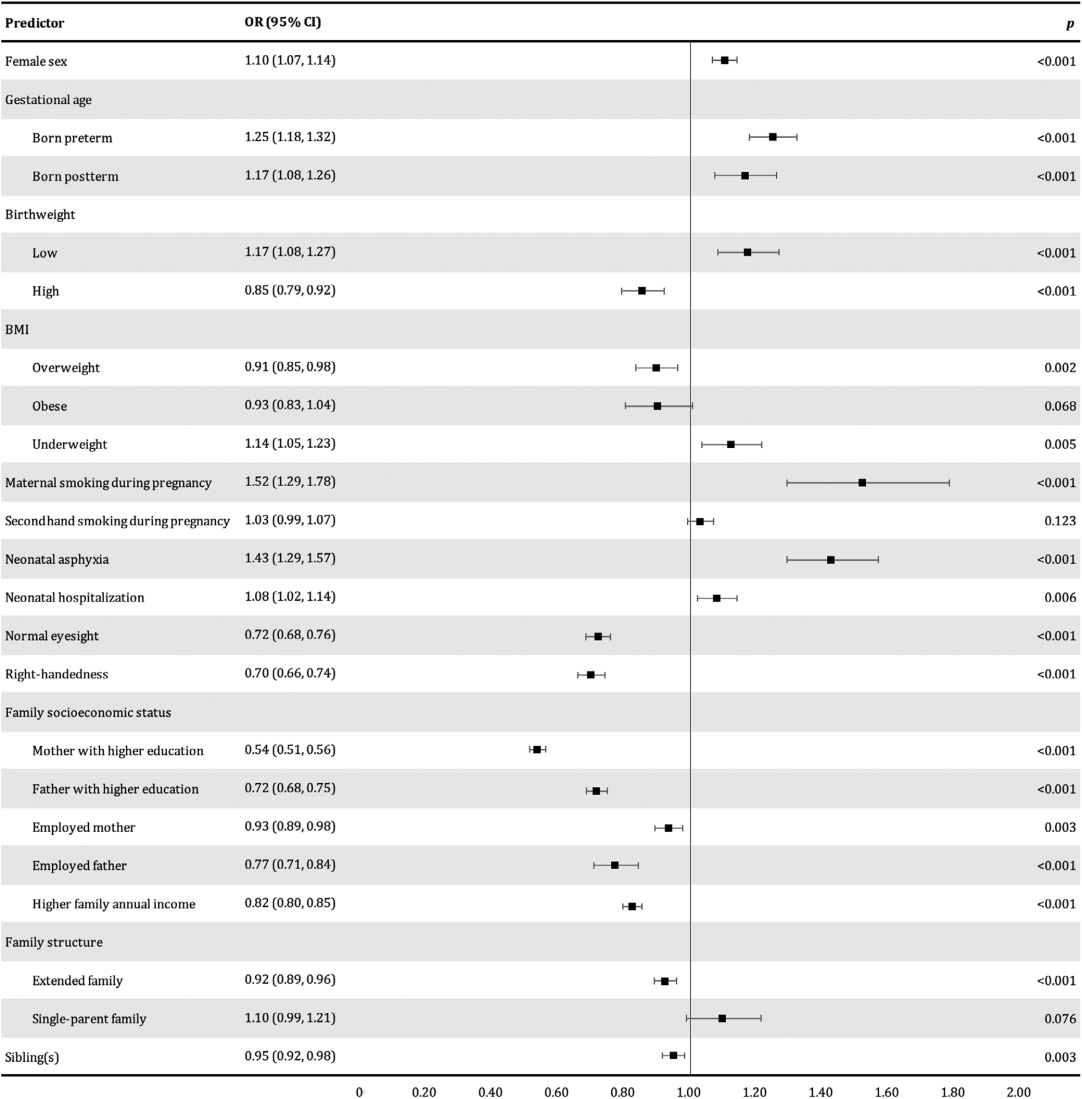
Association between predictors and possible developmental coordination disorder (*n* = 105 663). Abbreviations: BMI, body mass index; CI, confidence interval; OR, odds ratio.

Seventeen key indicators were identified as the final predictors in the model for the early prediction of DCD, including seven family‐level (mother with higher education, father with higher education, employed mother, employed father, higher family annual income, family structure, and sibling status) and 10 individual‐level (sex, birthweight, gestational age, maternal exposure to secondhand smoke, neonatal hospitalization, neonatal asphyxia, maternal smoking, BMI, vision status, and handedness) indicators (Figure 2).

### Feature contribution to prediction

**FIGURE 3:**
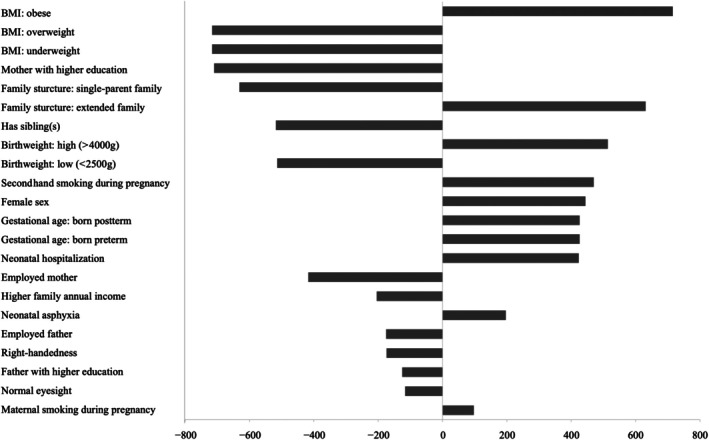
Variable importance ranking for predicting developmental coordination disorder: mean decrease in Gini index. Abbreviation: BMI, body mass index.

The hierarchy of variable importance in predicting possible DCD using the mean decrease in Gini index can be found in Figure 3. The top predictors were the child's BMI (mean decrease in Gini index = 715.67), mother with higher education (mean decrease in Gini index = 709.78), and family structure (mean decrease in Gini index = 631.00). Being underweight, single‐parent family, and low birthweight showed strong positive predictive relationships with a high risk of DCD.

### Internal validation

**FIGURE 4:**
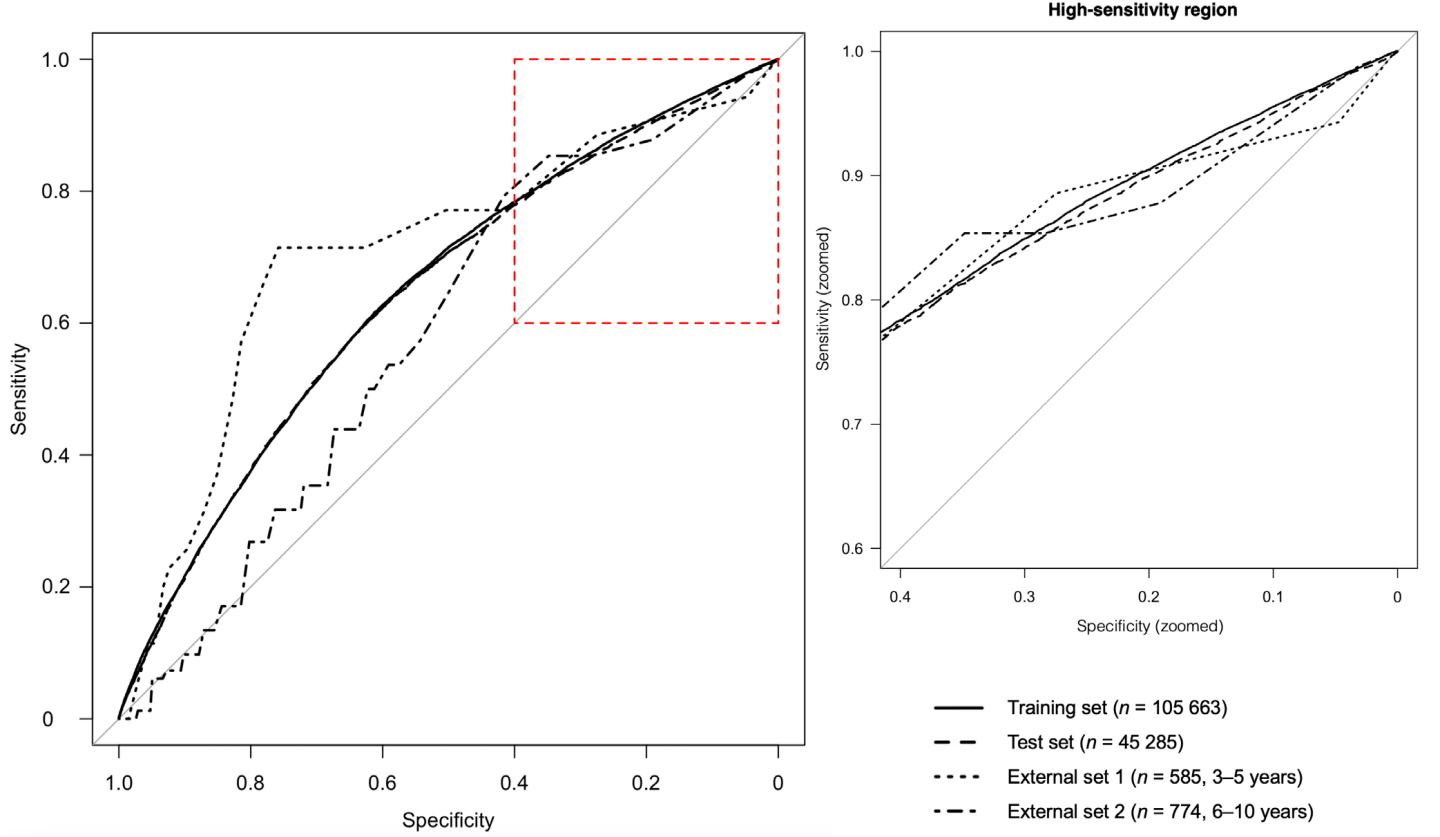
Receiver operating characteristic curves in each data set. The red dotted box indicates the zoomed high‐sensitivity region shown on the right.

In the training set, the predictive model discriminated between possible DCD and typical development, with an AUC of 0.65 (95% confidence interval [CI] = 0.64–0.65, *p* < 0.001), indicating moderate discrimination. The AUC in the testing set of the model was 0.64 (95% CI = 0.64–0.65, *p* < 0.001), closely aligning with the training set, which suggests good generalization and consistent model performance with minimal overfitting (Figure 4).

### External validation

For the group aged 3 to 5 years, the model discriminated between DCD and typical development with an AUC of 0.70 (95% CI = 0.61–0.80, *p* < 0.001) (Figure 4), a sensitivity of 71.43%, accuracy of 77.61%, and specificity of 78.00% (Table 1), indicating a moderate‐to‐high performance for DCD diagnosis.

**TABLE 1:** External validation of the model performance metrics.

Performance metric	Age group	
	Aged 3–5 years (*n* = 585)	Aged 6–10 years (*n* = 774)
Performance indicator, %		
Sensitivity	71.43	54.88
Accuracy	77.61	61.50
Specificity	78.00	62.28
Prediction outcome, *n*		
True positive	25	45
False positive	121	261
True negative	429	431
False negative	10	37

In the group aged 6 to 10 years, the model's AUC was 0.58 (95% CI = 0.52–0.64, *p* < 0.01) (Figure 4), sensitivity was 54.88%, accuracy was 61.50%, and specificity was 62.28% (Table 1), reflecting a moderate but lower performance in DCD prognosis.

## DISCUSSION

This study presents a significant advancement in the early screening of DCD by developing and externally validating a screening model that goes beyond the traditional focus on observable motor performance deficits. Unlike conventional approaches, our model integrates a broad spectrum of risk factors, including maternal health conditions and family environmental variables, which are normally readily available in health records in many countries, although accessibility may be limited in regions with a less developed digital health infrastructure. This approach offers significant reductions in time and resources by minimizing dependence on performance‐based screenings that primarily rely on caregiver observations, to accelerate the pathway to diagnosis and intervention. The robustness of our model is supported by rigorous internal and external validation processes, which have demonstrated its high sensitivity and specificity.

Recent advances in understanding the risk factors of DCD provided a unique opportunity to integrate current evidence to identify children at higher risk according to their routine health examination data. It is important to note that our study was not focused on diagnosing existing DCD or to discover new risk factors. Instead, we focused on integrating established perinatal, family, and individual sociodemographic risk factors into a coherent clinical prediction algorithm. The risk factors in our model were selected based on theoretical frameworks and the previous literature, and were confirmed using machine learning technology. Our model considers both immutable factors, such as sex and perinatal health factors, and mutable factors like BMI, thus providing a comprehensive bio‐ecological perspective on DCD. The elements used in our model are standard in clinical and community health records, enabling easy integration into existing health systems. In practice, this model helps paediatricians and health workers identify at‐risk children early for prompt screening and intervention, regardless of clear motor signs. For policy, it can integrate into routine health checks to proactively identify risks, ensure fair access to services for high‐risk populations, and optimize resource use.

The application of our predictive model across a diverse age spectrum is crucial, particularly given the challenges of screening DCD in its early stages. Those identified by the model as being at high risk can then be referred to health care professionals for further clinical diagnosis. Our model showed substantial predictive accuracy in the group aged 3 to 5 years, achieving an AUC of 0.70, sensitivity of 71.43%, accuracy of 77.61%, and specificity of 78.00%. This efficacy in early detection underscores the model's capability to identify potential DCD during a pivotal developmental phase, when traditional screening methods often prove inadequate. Furthermore, our model retains its utility in older children aged 6 to 10 years, demonstrating an AUC of 0.58, sensitivity of 54.88%, accuracy of 61.50%, and specificity of 62.28%. Despite a slight decline in performance metrics compared to the younger cohort, these results are still meaningful and highlight the model's extended applicability across a broader age range. This is especially significant because the manifestations of DCD and the influence of its risk factors can vary and intensify with age. The model's consistent performance across these critical age groups indicates its effectiveness in capturing both persistent and emerging risk factors. This capability is vital for formulating intervention strategies that are both timely and flexible, adapting to the evolving needs of growing children. By enabling early risk identification and intervention, the model provides a proactive approach to managing DCD, potentially enhancing opportunities for children to receive timely clinical diagnoses and interventions. Thus, our model supports a more inclusive and effective approach to early screening and intervention, potentially improving long‐term outcomes for children at risk of DCD.

By prioritizing high specificity, we aim to reduce the burden on health care providers by minimizing false positives. Importantly, this model is not intended for diagnosis or labelling but serves as a pre‐screening tool to flag children who may benefit from further developmental assessment. It is essential to interpret the model's outputs as indicators of general developmental risk rather than definitive diagnostic labels. Moreover, the model was not designed to distinguish DCD from other neurodevelopmental disorders, such as autism spectrum disorder or attention‐deficit/hyperactivity disorder, which frequently co‐occur and can share similar early risk factors and motor profiles. Although our development and validation samples excluded children with known severe neurodevelopmental conditions, the possibility of undetected comorbidities cannot be fully ruled out. Children identified as being at high risk should undergo comprehensive, multidisciplinary evaluation before any clinical decisions are made. We emphasize that the model is intended as a pre‐screening tool to support early triage and does not substitute professional diagnostic judgement or structured motor assessments. This is particularly important in health care systems with limited resources, where inappropriate reliance on algorithmic screening could lead to misallocation of services or unintended parental anxiety.

In addition, we recommend adaptable cut‐off points that can be adjusted based on specific community health profiles and local health care resources. Although the model was developed using a Chinese national sample, it incorporates universally relevant risk factors, such as perinatal health and family dynamics. To apply the algorithm in contexts beyond the initial Chinese cohort, future research should explore the utility of the average effect estimates derived from our model and investigate methods for tailoring it according to regional data that closely match the sociodemographic characteristics of our study population. This approach ensures that the algorithm remains robust and applicable across diverse populations, thereby enhancing its utility in clinical settings worldwide. In practice, our model enables health care systems to flag children at increased risk for DCD in their databases, prompting timely diagnostic assessments. Previous studies indicated that many children with symptoms of DCD go undiagnosed for considerable periods, with an average delay of 30 months from initial help‐seeking to actual diagnosis.^22^ Early identification through our model could help to reduce this delay significantly, potentially lowering the long‐term social and economic costs associated with untreated DCD. Although our model lays a solid groundwork for risk identification, the real‐world impact of early intervention based on these predictions needs further investigation. Future studies should focus on validating the model's effectiveness across diverse health care environments to ensure its adaptability and efficacy.

By drawing on the bio‐ecological model and the International Classification of Functioning, Disability, and Health framework, our model captures a wide range of early‐life risk factors, offering a scalable and accessible tool to support earlier identification in non‐clinical settings. Our findings suggest that such an approach can help address delays in referral and diagnosis, particularly in resource‐limited environments where standardized behavioural assessments are not routinely available. However, as with all predictive tools, the use of predictive models in developmental screening must be approached with caution. In deploying a predictive model for developmental risk, ethical considerations must be emphasized. Risk estimates should be communicated carefully to avoid unnecessary anxiety or misinterpretation. Importantly, follow‐up decisions must continue to rely on professional clinical diagnosis and judgement. The model is designed to support, not replace, existing diagnostic pathways and should be interpreted within the broader developmental and contextual profile of the child. Ensuring that predictive outputs are used responsibly, particularly in discussions with caregivers, is essential.

### Strengths and limitations

This study has several strengths. First, the use of a bio‐ecological framework, incorporating multiple domains of risk factors such as maternal health, family environment, and perinatal influences, offers a comprehensive approach to DCD screening. Additionally, the study's rigorous internal and external validation across different age groups further confirms the model's robustness and applicability. Specifically, the model has been tested not only in children aged 3 to 5 years, a critical period when early identification and intervention can have a substantial impact on developmental outcomes, but also in children aged 6 to 10 years. This allows for broader applicability and demonstrates the model's capacity to track the consistency and accuracy of risk prediction across a wider developmental spectrum. Moreover, the inclusion of predictors that are routinely available in clinical and community health care records, such as maternal health, family socioeconomic status, and early childhood conditions, makes the model practical for integration into existing health care systems. This accessibility facilitates early risk stratification without imposing additional burdens on health care providers or requiring specialized data collection efforts.

While our study provides significant insights into early detection of DCD using a bio‐ecological model, it is not without limitations. First, although our model incorporates a range of theoretically and empirically informed risk factors, there are potential other influential variables; genetic predisposition, detailed measures of environmental quality, and early developmental milestones were not available in our data set. Second, while the model was validated in both younger (3–5 years) and older (6–10 years) age groups, its long‐term predictive accuracy and effectiveness in different health care settings require further exploration. Moreover, the model was developed and tested within a single national context; its generalizability to other health care systems, populations, and cultural settings has yet to be confirmed. Future research should prioritize external validation in diverse international contexts and explore the need for local recalibration to reflect varying epidemiological patterns, data infrastructure, and service delivery models.

### CONCLUSIONS

Our study developed and externally validated a new bio‐ecological predictive model using readily available clinical record data for early screening of DCD among preschool children, leveraging comprehensive risk factors from electronic health records. This model was validated through rigorous internal and external assessments, demonstrating high sensitivity and specificity, and offering a cost‐effective and time‐efficient solution that could be easily integrated into existing health care frameworks. While further validation in different settings is essential, our findings suggest that this model could transform early DCD identification practices, reducing both time and financial burdens on health care systems.

## Supporting information


**Appendix S1:** Model card for papers involving artificial intelligence/machine learning

**Table S1:** Variable definitions and reference levels

**Table S2:** General characteristics in the four data sets

## Data Availability

The data that support the findings of this study are available on request from the corresponding author. The data are not publicly available due to privacy or ethical restrictions.
