# Supplementary material for: A bio‐ecological model for early screening of developmental coordination disorder
Source: Dev Med Child Neurol. 2025 Oct 3;68(6):774–83. doi: 10.1111/dmcn.70000 (PMC13160394; doi:10.1111/dmcn.70000)
Supplement: Supplementary file 1 — Appendix S1: Model card for papers involving artificial intelligence/machine learning [file DMCN-68-774-s003.pdf]

DMCN Developmental Medicine & Child Neurology

Mac Keith Press

Model Card for papers involving artificial intelligence/machine learning

Please provide details about the model used in the study in order to present the model synthetically for the benefit of the readership and allow reproducibility.

Model details

Aim of the model	To develop a risk prediction model for Developmental Coordination Disorder (DCD) in early childhood using routinely collected health
Architecture/algorithm type	Logistic regression with stepwise selection (guided by AIC), supported by random forest for variable importance ranking.
Model input	Model input involved 21 candidate predictors, resulting in a final set of 17 predictors selected across sociodemographic, maternal, and individual factor categories
Model output	Binary classification: predicted probability of being at risk for DCD.
Hyperparameters	Not applicable for logistic regression; random forest used default parameters in scikit-learn (n_estimators = 100, max_depth = None).
Preprocessing steps	Direct data extraction from a complete and well-developed system (no missing data processing was required); Single 70-30 train-test split; Regression model development using

Software environment and code availability

Link to source code (if not available, please justify)	Not publicly available due to institutional restrictions, but available upon reasonable request.
Software and library versions	R Statistical Software (version 4.3.3, 2024-02-29)
Hardware requirement	No specific hardware requirements

Data sources

Link to data sources (if sharing the data is not possible, authors should describe how data were collected)	Data from the Chinese National Cohort of Motor Development (CNCMD); not publicly available due to privacy and ethics policies,but available upon reasonable request. Participants were recruited through a national stratified cluster sampling design.
Description of data set	Development sample: 150,948 children aged 3–5 years; External validation sample: 1,359 clinically assessed children aged 3–10 years

Evaluation metrics and results

Description of evaluation metrics	Area under the ROC curve (AUC), sensitivity, specificity, accuracy
Description of evaluation methodology (training set; validation/test set)	Data randomly split: 70% training, 30% internal test set; External validation performed on an independent clinical dataset; No cross-validation due to large sample size ($n > 150,000$).
Model performance and statistical analysis	Internal training set: AUC = 0.65 ($p < 0.001$); Internal test set: AUC = 0.64 ($p < 0.001$) External 3–5 years subgroup: AUC = 0.70, sensitivity = 71.43%, accuracy = 77.61%, specificity = 78.00%; External 6–10 years
